# Dexamethasone-Activated MSCs Release MVs for Stimulating Osteogenic Response

**DOI:** 10.1155/2018/7231739

**Published:** 2018-04-16

**Authors:** Mingyan Zhao, Peng Li, Haijia Xu, Qunwen Pan, Rong Zeng, Xiaotang Ma, Zhanghua Li, Hao Lin

**Affiliations:** ^1^Stem Cell Research and Cellular Therapy Center, Affiliated Hospital of Guangdong Medical University, Zhanjiang 524001, China; ^2^Department of Orthopaedics, Tongren Hospital of Wuhan University, Wuhan 430060, China; ^3^Department of Surgery, Guangdong Key Laboratory of Age-Related Cardiac and Cerebral Diseases, Affiliated Hospital of Guangdong Medical University, Zhanjiang 524001, China; ^4^Department of Spinal Surgery, Affiliated Hospital of Guangdong Medical University, Zhanjiang 524001, China

## Abstract

The extracellular microvesicles (MVs) are attracting much attention because they are found to be the key paracrine mediator participating in tissue regeneration. Dexamethasone (DXM) is widely accepted as an important regulator in tailoring the differentiation potential of mesenchymal stem cells (MSCs). However, the effect of DXM on the paracrine signaling of MSCs remains unknown. To this point, we aimed to explore the role of DXM in regulating the paracrine activity of MSCs through evaluating the release and function of MSC-MVs, based on their physicochemical characteristics and support on osteogenic response. Results showed that DXM had no evident impact on the release of MSC-MVs but played a pivotal role in regulating the function of MSC-MVs. MVs obtained from the DXM-stimulated MSCs (DXM-MVs) increased MC3T3 cell proliferation and migration and upregulated Runt-related transcription factor 2 (Runx2), alkaline phosphatase (ALP), and osteopontin (OPN) expression. The repair efficiency of DXM-MVs for femur defects was further investigated in an established rat model. It was found that DXM-MVs accelerated the healing process of bone formation in the defect area. Thus, we conclude that using DXM as stimuli to obtain functional MSCs-MVs could become a valuable tool for promoting bone regeneration.

## 1. Introduction

Mesenchymal stem cells (MSCs) are multipotent stem cells with the capability to differentiate into osteoblasts, adipocytes, chondrocytes, and myoblasts [1]. Owing to their regenerative potency, MSCs attract considerable interest in clinical applications for the treatment of wide spectrum of diseases. However, with the increased utilization and scrutiny of MSCs, the initial differentiation-based rationale for their application has become gradually untenable [2, 3]. Recent studies reported that the paracrine activity of MSCs has great effects on their therapeutic efficiency towards a variety of diseases including tissue injury in lung, skeletal muscle, liver, and kidney [4–7].

On the other hand, microvesicles (MVs), one type of extracellular vesicles (EVs), have gained considerable interest as novel mediators in cell-to-cell communication. MVs are submicron membrane vesicles released by numerous types of cells in response to different stimuli [8]. They carry a battery of signaling molecules such as mRNA and microRNA (miRNA) as well as proteins and serve as a vehicle to transfer these messages to neighbor and distant cells to modulate the proliferation and differentiation of recipient cells [9–11]. The properties of MVs represent the particular characteristics of their original cells and the environment. Emerging evidence suggests that environment stimuli greatly influence the paracrine signaling of parent cells and thereby regulate the responses of recipient cells [12–14].

Dexamethasone (DXM) is a potent synthetic form of the steroid glucocorticoid that has been widely used in a variety of medical and biological applications. Clinically, DXM has been utilized as an anti-inflammatory drug [15]. Previous studies reported that DXM can induce osteoporosis and even pathological fracture [16], while DXM in vitro promotes osteoblast differentiation and bone mineralization [17–19]. Actually, DXM has been routinely used to induce the differentiation of MSCs and is a key component in osteogenic differentiation medium. Nevertheless, differential effects of DXM on undifferentiated MSCs and osteoblasts have been reported [20]. Specifically, low DXM concentration enhances MSC commitment and promotes differentiation while high concentrations and long-term treatments suppress the maturation and terminal differentiation of osteoblasts [21–23]. The typical DXM concentration of 100 nM induces osteogenesis [24], while the high concentration leads to adipogenic differentiation [21, 24].

Since DXM is an important mediator in regulating the biological responses of MSCs, it is interesting to know whether the DXM could impact the paracrine signaling of MSCs. Here, we investigated the effect of DXM on the release of MSC-MVs and the influence of MSC-MVs in osteogenic healing in *in vitro* and *in vivo* experiments.

## 2. Materials and Methods

### 2.1. Cell Culture

Rat bone marrow-derived mesenchymal stem cells (MSCs) were obtained from Cyagen (Guangzhou, China) and cultured in Dulbecco's modified Eagle's medium. MC3T3-E1 preosteoblasts were purchased from the Cell Bank of the Chinese Academy of Sciences (Shanghai, China) and grow in alpha modified Eagle's medium (*α*MEM, Gibco, Grand Island NY, USA), supplemented with 10% fetal bovine serum (FBS, Gibco) and 1% antibiotic-antimycotic solution (Gibco). Cells were cultured in a 37°C incubator with a humidified 5% CO_2_/95% air atmosphere.

### 2.2. Isolation and Characterization of Extracellular Microvesicles (MVs)

MVs were harvested from the medium of MSCs cultured in the normal medium (n-MVs) or in DXM (Sigma, St. Louis, MO, USA) medium (DXM-MVs). Briefly, MSCs (passage 4–6) were seeded in T-75 cell culture flaks. At 80% confluence, cells were washed once with sterile PBS (pH 7.4, Gibco) and grown in fresh normal growth medium or DXM-supplemented medium (at a concentration of 10^−6^, 10^−7^, or 10^−8^ M) for 48 hrs. Then, MVs were isolated according to a protocol previously published [13]. In brief, the cell culture medium was collected and centrifuged at 300*g* for 15 min, followed by 2000*g* for 30 min to remove the cell debris. After that, MVs were pelleted from the cell-free culture medium by centrifugation (Hitachi CS150GXII, Tokyo, Japan) at 20,000*g* for 2 hrs at 4°C.

Final MV pellets were resuspended in 200 *μ*L PBS. The concentration and size distribution of MVs were determined using the nanoparticle analyzer system (NTA300, Malvern, Britain). The surface markers of MVs were investigated by flow cytometry (BD FACSCantoII, San Jose, CA, USA) analysis as previously reported [25]. Briefly, MVs were resuspended and incubated with PE-conjugated CD90 or isotype-matched control for 30 min at room temperature in the dark. All antibodies were obtained from BD biosciences (San Jose, CA, USA).

To verify the ultrastructure, isolated MVs were fixed with 3% glutaraldehyde (Sinopharm Chemical Reagent Shanghai Co. Ltd, Shanghai, China) diluted in PBS. Five nanoliter of fixed MVs were dropped onto a Formvar carbon-coated grid and allowed to dry in a cabinet at 23°C for 20 min. After rinsing with PBS, the MVs were fixed in 1% glutaraldehyde for 5 min, followed by washing with distilled water and staining with saturated aqueous uranyl oxalate for another 5 min. Finally, the excess liquid was removed, and samples were dried and visualized by transmission electron microscopy (TEM, JEM-1400, JEOL, Tokyo, Japan).

### 2.3. Concentration-Response Study of MSCs Derived MVs (MSC-MVs) on MC3T3 Viability

To determine the dose-effect of MSC-MVs on MC3T3 viability, cells were treated with different doses (0, 10^6^, or 10^7^ mL^−1^) of MSC-MVs. After 24 hrs of coculture, the cell viability was assessed using a MTS kit (Promega, Madsion, USA). For the assay, the medium was carefully aspirated, and cells were rinsed once with sterile PBS (Gibco). Then, 120 *μ*L of *α*MEM supplemented with MTS assay reagent (ratio of 5 : 1) was added into each well and incubated for another 2 hrs. Finally, the measurement of MTS adsorption was performed at 490 nm using a 96-well plate reader (Thermo Fisher, Waltham, MA, USA).

### 2.4. In Vitro Function Assays

#### 2.4.1. Detection of MSC-MVs Merging with MC3T3

To determine whether MSC-MVs could be captured and internalized by MC3T3, a lipid membrane-intercalating fluorescent dye PKH26 (sigma) was used to label MVs before coculture according to manufacturer's instructions with some modifications. Briefly, MVs were mixed with 2 *μ*M PKH26 at room temperature for 5 min. Thereafter, the staining was stopped by adding an equal volume of 1% bovine serum albumin (BSA, GenDEPOT, Barker TX, USA). The PKH26-labeled MVs were obtained by ultracentrifuging and then added to MC3T3 in culture medium. After 24 hrs of incubation, the cells were washed three times in PBS and then fixed with 4% paraformaldehyde (sigma) for 10 min. The cell nuclei were stained by DAPI (Invitorgen, USA). The interaction between MSC-MVs and MC3T3 was examined and photographed using confocal laser scanning microscopy (CLSM, Leica TCS SP5II, Wetzlar, Germany). Images were processed with the Leica LAS AF Lite (Leica).

#### 2.4.2. MC3T3 Proliferation Assay

Based on the above study, 10^7^ mL^−1^ of MSC-MVs were used in the subsequent experiments. The proliferative capability of MC3T3 treated with n-MVs or DXM-MVs was tested using MTS assay kit after 1, 2, and 3 days of culture. The MC3T3 grown in the normal growth medium was set as control. The details of the measurement are the same as described above.

#### 2.4.3. Migration Activity of MC3T3

The effect of MSC-MVs on the migration of MC3T3 was assessed by a scratch study, as previously reported [26]. MC3T3 cells were seeded on 6-well plate (NEST, Shanghai, China) and cultured for overnight to reach confluence. Then, a scratch was made through the cell monolayer using a P200 pipette tip. After carefully washing with PBS twice, cells were cultured in growth medium supplemented with n-MVs or DXM-MVs. The invasion of cells into the scratch area was monitored by taking images immediately (0 h) and 12 and 20 hrs after making the scratch. Quantitative analysis of cell migration was evaluated from five images per sample according to the following calculation:

(1) $$\begin{array}{c} \frac{\left(Width\text{ of }injury\;line\;at\;0\;h-width\text{ of }injury\;line\;at\;20\;h\right)}{Width\text{ of }injury\;line\;at\;0\;h}\times 100\%. \end{array}$$

#### 2.4.4. Culture of MC3T3 for Osteogenesis

The MC3T3 cells were seeded at 5 × 10^4^ cells/well in a 24-well plate or 2.5 × 10^5^ cells/well in a 6-well plate. To examine the effects of MSC-MVs on osteogenic differentiation, the medium was changed to *α*MEM, n-MV suspension in *α*MEM, or DXM-MV suspension in *α*MEM containing 1% FBS and 1% penicillin-streptomycin. The cells cultured in the osteogenic differentiation medium (OM) consisted of basal medium (BM, 1% FBS, and 1% penicillin-streptomycin-containing *α*MEM) supplemented with osteogenic supplements, namely, 10 nM dexamethasone (Sigma), 50 mg mL^−1^ ascorbic acid (Sigma), and 10 mM *β*-glycerophosphate (sigma), were used as positive control. The medium was changed every 3 days.

#### 2.4.5. Detection of Osteogenic Differentiation of MC3T3

Calcium phosphate deposition was investigated by alizarin red staining on day 21 postdifferentiation. Briefly, the cells were washed once with PBS and fixed in 4% paraformaldehyde for 10 min. After washing with distilled water twice, samples were stained with 2% alizarin red S solution (pH 4.2, Cyagen) for 45 min in the dark at room temperature. Finally, the excess dye was removed by thoroughly rinsing with distilled water. Images were captured using an EVOS™ XL Core Cell Imaging System (Thermo Fisher Scientific).

The expression levels of osteogenic genes including Runt-related transcription factor 2 (Runx2), alkaline phosphatase (ALP), and osteopontin (OPN) were evaluated by quantitative real-time (qRT-PCR) to investigate the differentiation extent of MC3T3 towards osteogenic lineage. On day 7 and day 14 postdifferentiation, cells were washed once with PBS and harvested. Total RNA was extracted via RNAiso Rlus (Takara, Shiga, Japan) and then transcribed into cDNA using PrimeScriptTM RT Master Mix Kit (Takara) based on manufacturers' protocols. The SYBR Premix Ex TaqTM II (Takara) were used for qRT-PCR. Reactions were performed on a LightCycler480 Real-time PCR System (Roche, Basel, Switzerland) according to the following protocol: 95°C for 5 min, 40 cycles of 95°C for 15 second, 60°C for 15 second, and 72°C for 30 second. The expression of mRNA in the cells was assessed by the use of GAPDH as the endogenous control. The primers were designed and presented in Table 1. The data were analyzed by 2^^-ddct^ method [27].

### 2.5. In Vivo Bone-Healing Experiments

#### 2.5.1. Animal Model

Based on the in vitro function studies, 10^−7^ M DXM-activated MSC-derived MVs (DXM-MVs) were used in the subsequent experiments. Female SD rats were purchased from the Guangdong Medical Laboratory Animal Center. All animals were randomly divided into model group and DXM-MV group. All animal experiments were approved by the Laboratory Animal Care and Use Committees at Guangdong Medical University and met the National Institutes of Health guidelines for the care and use of laboratory animals. To study the effect of the DXM-MVs on bone repair, a 2 mm diameter and 1 mm depth femur defect was generated in each SD rat. Holes were extensively rinsed with saline to remove bone fragments from the cavity. Subsequently, the DXM-MV group was treated by injection of 5 × 10^7^ mL^−1^ DXM-MVs in PBS into the defect area, while the model group was treated by injection of equal volume of PBS. On week 2, 4, and 6 of postoperation, bone regeneration in the defect area was evaluated and further assessed by histological techniques, as described below in details.

#### 2.5.2. In Vivo X-Ray Imaging

To determine the bone mineral density (BMD), X-ray images of the tested rats were captured using a Bruker Xtreme Imaging System (Bruker, Billerica, MA, USA) at selected time points (0, 2, and 4 weeks). X-rays were collected under an exposure time of 10 s, with the f-stop 2.0 mm and FOV 160.0 mm. The vertical and horizontal resolutions were 773 ppi with X-ray energy 45 KVP. Recorded images were processed and analyzed by the Bruker molecular imaging software (Bruker).

#### 2.5.3. Micro-CT Imaging

CT images of the right femur were acquired using a micro-CT scanner (La Theta LCT200; Aloka, Tokyo, Japan) following manufacturer's protocol. Parameters including cancellous, cortical, and total BMD were calculated using the La Theta software (version 3.00). Cancellous bone volume was calculated from the term “trabecular volume,” as defined by the software, which refers to the cancellous volume excluding bone marrow volume. Total bone volume was calculated from the term “subtotal volume,” as defined by the software, which designates as the volume of cortical and trabecular bone.

#### 2.5.4. Histological Analysis

On week 4 and 6 postoperation, three rats from each group were sacrificed under general anesthesia with intraperitoneal injection of 1% pentobarbital sodium (0.1 mL/100 g, Solarbio, Beijing, China). The femurs were harvested and processed following a previously published protocol [28]. Briefly, the isolated femurs were fixed in 10% neutral-bufferd formalin for 24 hrs and then immersed into 9% formic acid for decalcification. Thereafter, the specimens were dehydrated and embedded in paraffin. Several sections (5 *μ*m) were cut along with the long axis of femour shaft and collected on glass slides for hematoxylin and eosin (H&E, Solarbio) and ALP staining (Solarbio) using standard protocols. After mounting with coverslips, the sections were observed and analyzed by an optical microscope.

### 2.6. Statistical Analysis

All quantitative data are expressed as mean ± standard deviation (SD). Data were analyzed by Student's *t*-test or one-way ANOVA followed by post hoc Tukey testing using Origin software (Originlab Corporation, Northampton, USA). A *P* value of lower than 0.05 was considered to be statistically significant.

## 3. Results

### 3.1. Characterization of MSC-MVs

**Figure 1:**
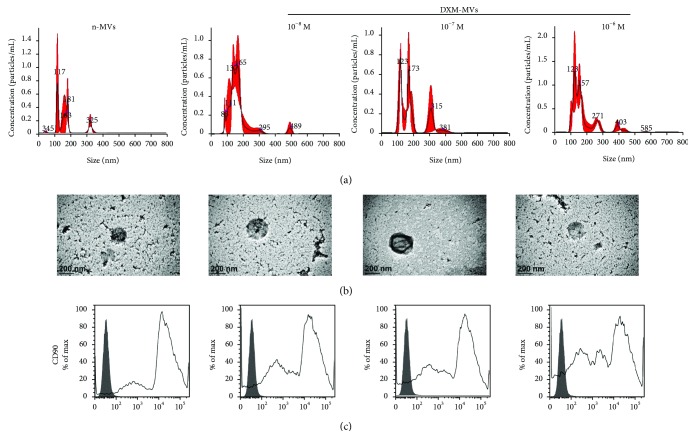
Characterization of MSC-MVs (n-MVs or DXM-MVs). MSCs were activated by treatment with 10^−8^, 10^−7^, and 10^−6^ M DXM. (a) Size distribution and concentration of MSC-MVs detected by NTA analysis. (b) Typical morphology of MSC-MVs investigated by a transmission electron microscope. (c) Flow cytometric analysis showing the expression of MSC-specific marker CD90 in MSC-MVs.

The size distribution and concentration of MSC-MVs were detected by NTA analysis (Figure 1(a)). It was found that the addition of DXM had no evident effect on the release of MVs, where the size distribution of BMSC-MVs was ranged from 100 nm to 400 nm with the concentration around 2 × 10^8^ mL^−1^. The ultrastructure of MSC-MVs investigated by TEM (Figure 1(b)) shows that the MVs derived from the four different culturing conditions of MSCs exhibited a spheroid shape with a diameter about 200 nm. Flow cytometric analysis revealed that MSC-MVs expressed MSC-specific marker CD90 (Figure 1(c)). Together, these results suggested that MVs were successfully isolated from MSCs.

### 3.2. Effect of MSC-MVs on MC3T3 Viability

**Figure 2:**
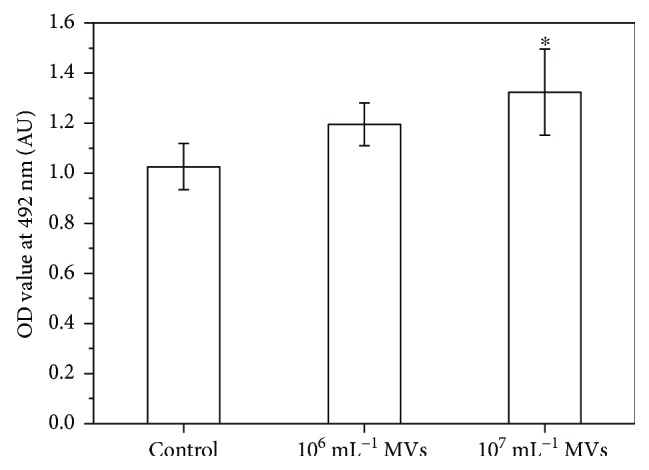
The effect of MSC-MV concentration on MC3T3 viability. ^∗^*P* < 0.05 versus control, *n* = 5.

To determine the effect of MSC-MVs on MC3T3 viability, different doses of MSC-MVs (0, 10^6^, and 10^7^ mL^−1^) were added into the culture medium and coincubated with MC3T3 for 24 hrs. It was found that MSC-MVs at the concentration of 10^7^ mL^−1^ enhanced the viability of MC3T3 (Figure 2). Thus, we chose 10^7^ mL^−1^ for the following experiments.

### 3.3. MSC-MV Uptake by MC3T3 cells

**Figure 3:**
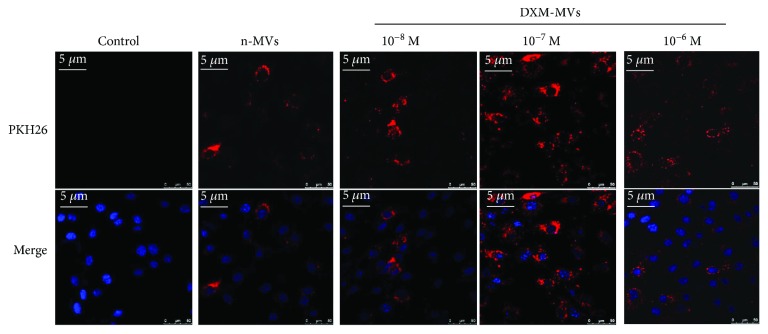
Uptake of MSC-MVs by MC3T3. PKH26-labelled (red) MVs or control was added to MC3T3 for coculture, and the interaction was investigated after coincubation for 24 hrs using CLSM. Representative images show that MSC-MVs merged with MC3T3. Nucleuses were stained with DAPI (blue).

To examine whether MSC-MVs interact with MC3T3, PKH26-labelled MVs were incubated with MC3T3 for 24 hrs and investigated under CLSM. The colocalization of fluorescence-labelled MVs and MC3T3 could be found in all the groups, indicating that the MVs could merge with MC3T3 (Figure 3). However, the number of cells positive for the stained MVs differed greatly among the samples. In the DXM-MVs (10^−7^ M) group, most of the cells were positive for the PKH26-labelled MVs, while only part of the MC3T3 was positive in the other groups.

### 3.4. DXM-MVs Dose-Dependently Promoted the Proliferation of MC3T3 at Lower Concentrations

**Figure 4:**
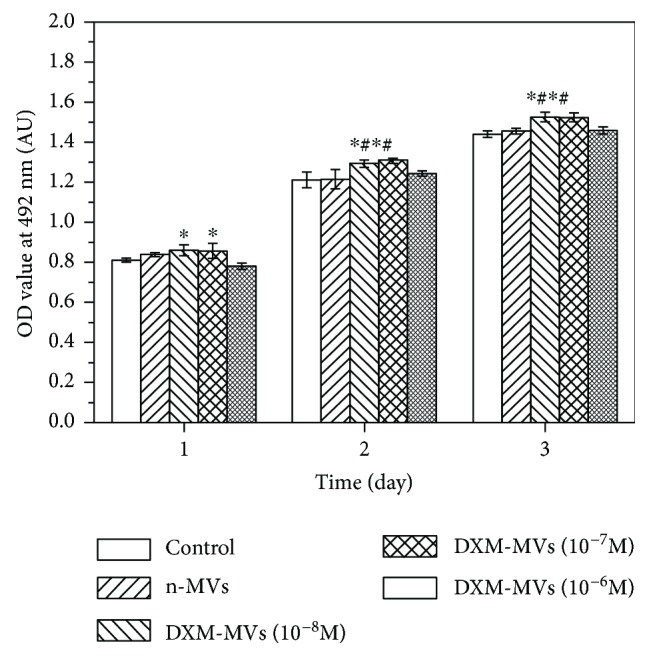
The effect of n-MVs and DXM-MVs on the proliferation of MC3T3 assessed by MTS assay at days 1, 2, and 3. ^∗^*P* < 0.05 versus control, ^#^*P* < 0.05 versus n-MVs, *n* = 3.

The proliferation of MC3T3 over a period of 1, 2, and 3 days treated with n-MVs or DXM-MVs was investigated by MTS assay which determines the metabolic activity of cells (Figure 4). The metabolic activity of cells treated with DXM-MVs was dose-dependent at relative lower DXM concentrations (*P* < 0.05). However, the DXM-MVs derived from relative higher DXM-activated MSCs (10^−6^ M) had no obvious impact on the metabolic activity of MC3T3 when in comparison to the n-MV group. The data in Figure 3 are consistent with the data in Figure 4.

### 3.5. DXM-MVs Increased the Migration Activity of MC3T3

**Figure 5:**
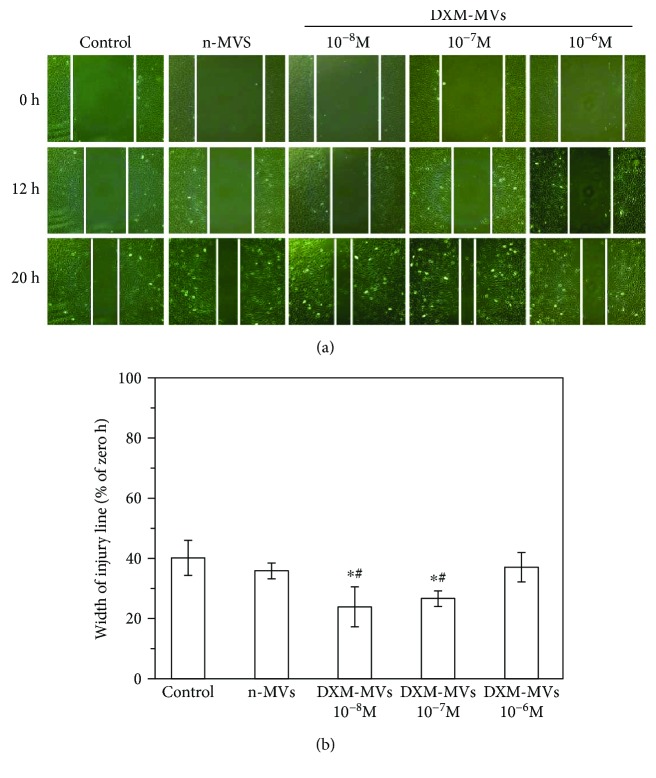
The effect of n-MVs and DXM-MVs on the migration activity of MC3T3. (a) Representative images of cell migration. (b) The quantitative analysis of cell migration activity in each group. Cell migration was measured by the scratch test, and wound closure was monitored by photographing at 0, 12, and 20 hrs of treatment of each compound. Cell migration (%) was quantified by calculating the wound width. ^∗^*P* < 0.05 versus control, ^#^*P* < 0.05 versus n-MVs, *n* = 12.

To investigate the effect of MSC-MVs on the migration activity of MC3T3, a scrape injury assay was performed. In consistent with the cell proliferation results, a pronounced difference in migration activity was found between n-MVs- and DXM-MV-treated group. The DXM-MVs of 10^−8^ and 10^−7^ M DXM-treated MSCs greatly increased the migration activity of MC3T3 as compared to the n-MV group (Figure 5).

### 3.6. DXM-MVs Stimulated the Osteogenesis of MC3T3

**Figure 6:**
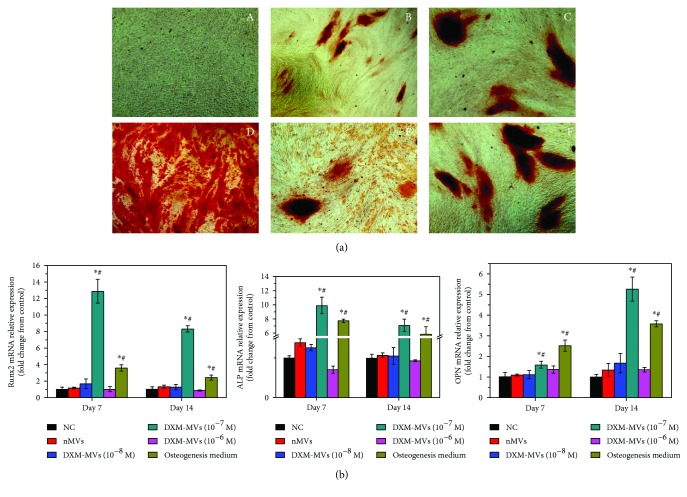
The effect of MSC-MVs on the osteogenic differentiation of MC3T3. (a) Histochemical staining of calcium phosphate deposition with alizarin red S at day 21 postdifferentiation (A: control, B: n-MVs, C: 10^−8^ M DXM-MVs, D: 10^−7^ M DXM-MVs, E: 10^−6^ M DXM-MVs, and F: osteogenesis medium). (b) The expression levels of Runx 2, ALP, and OPN at day 7 and day 14 postosteogenic differentiation measured by qRT-PCR. Relative gene expression is presented as normalized to gene expression in normal growth medium. ^∗^*P* < 0.05 versus control, ^#^*P* < 0.05 versus n-MVs.

The deposition of hydroxyapatite is one feature of mature osteoblasts. MC3T3 cultured under the different conditions were stained at day 21 postdifferentiation with alizarin red S to visualize calcium phosphate deposition (Figure 6(a)). No significant staining was observed when MC3T3 were cultured in normal growth medium while a staining was observed when cells were cultured in OM, indicating the need of osteogenic inductors for differentiation of these preosteoblast cells. However, it is of note that a positive staining was found on all MSC-MV-treated groups, although the staining differed greatly among the groups. Specifically, DXM-MVs (10^−7^ M) induced most extensive calcium deposition.

To further study the effect of MSC-MVs on the osteogenic differentiation of MC3T3, the osteogenic markers including Runx2, ALP, and OPN were determined by qRT-PCR at day 7 and day 14 postdifferentiation, respectively (Figure 6(b)). In line with the histochemical staining findings, DXM-MVs at the concentration of 10^−7^ M caused a significant upregulation of Runx2, ALP, and OPN expression in comparison to NC and n-MV groups. However, no significant differences were found among the other DXM-MV groups.

### 3.7. DXM-MVs Stimulated Bone Regeneration In Vivo

#### 3.7.1. The In Vivo Bone-Healing Process

**Figure 7:**
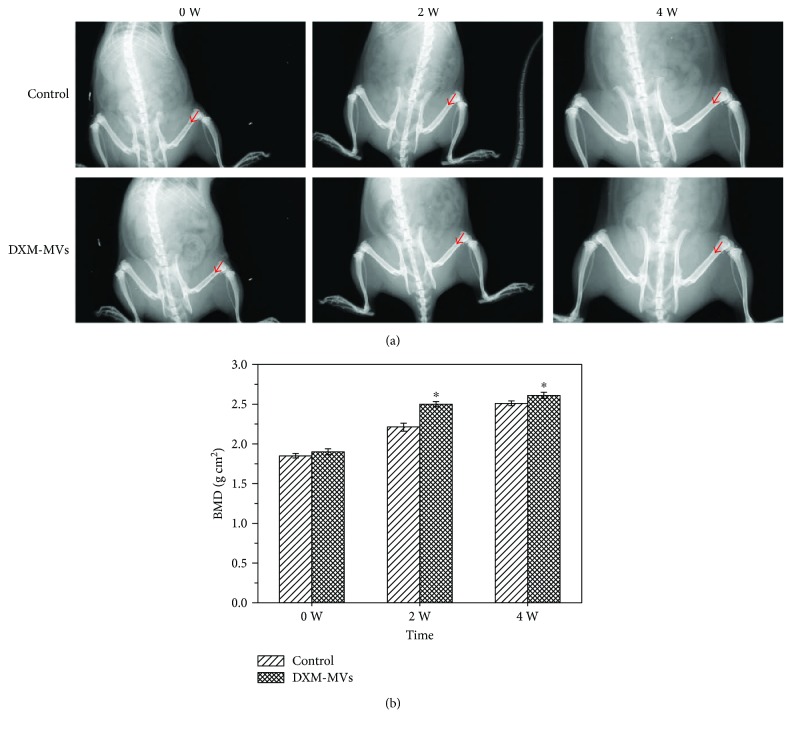
Bruker Xtreme in vivo imaging system monitoring the bone-healing process. (a) Representative pictures recorded by the system showing the bone-healing process at different time points postsurgery. (b) The quantitative analysis of BMD. ^∗^*P* < 0.05 versus control group, *n* = 5.

An in vivo imaging system was used here to monitor the bone-healing process. It was found that the bone defect area was gradually rebuilt with the increasing time (Figure 7(a)). There was no evident difference in BMD among the experimental groups on week 0 of postoperation. However, a significant difference was observed between the control and DXM-MV-treated rats on week 2 and 4 of postsurgery (Figure 7(b)).

#### 3.7.2. Micro-CT Analysis of New Bone Formation

**Figure 8:**
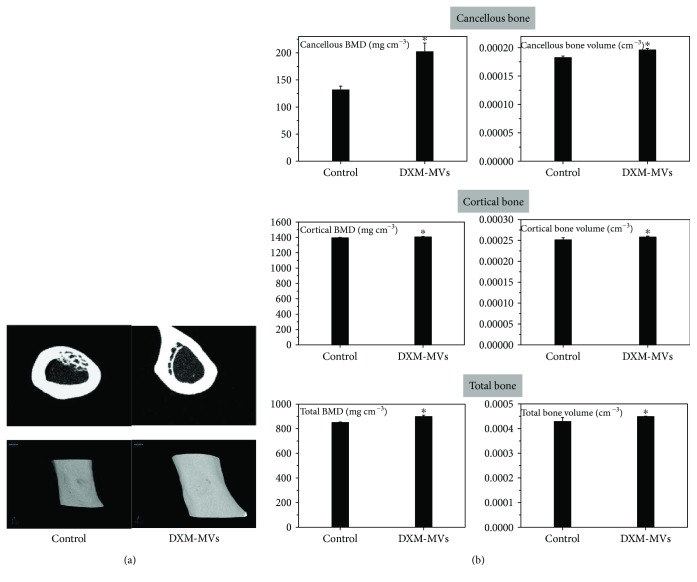
Micro-CT analysis of the effects of DXM-MVs on bone healing. (a) Micro-CT-generated images indicate the DXM-MVs accelerated the bone regeneration showing the largest amounts of new bone formation at 6 weeks after surgery. (b) The quantitative measurement of bone mineral density (BMD) and bone volume. ^∗^*P* < 0.05 versus control group, *n* = 6.

The new bone formation within the defects was investigated by micro-CT scan on week 6 postsurgery. The midpoint coronal plane 2D (Figure 8(a), upper panel) and 3D (Figure 8(b), lower panel) images were acquired and regenerated. It was found that DXM-MVs dramatically accelerated the bone regeneration and repair as demonstrated by a semiquantitative analysis (Figure 8(b)). As shown in Figure 8(a), the bone defects in DXM-MV-treated rats had almost completely reconstructed due to the newly formed calluses which were mostly mineralized based on the 2D coronal images. In contrast, the bone defects had not completely closed and the covered calluses had relative low density for the control group.

To quantify the new bone regeneration within the femur defects, the bone parameters including bone volume and BMD were measured. As shown in Figure 8(b), BMD and bone volume of the cortical bone and cancellous bone were significantly increased in DXM-MV-treated rats. Data indicated that the parameters of cortical and cancellous bone regeneration were greatly increased by DXM-MV treatment.

#### 3.7.3. Histological Analysis of Bone Healing

**Figure 9:**
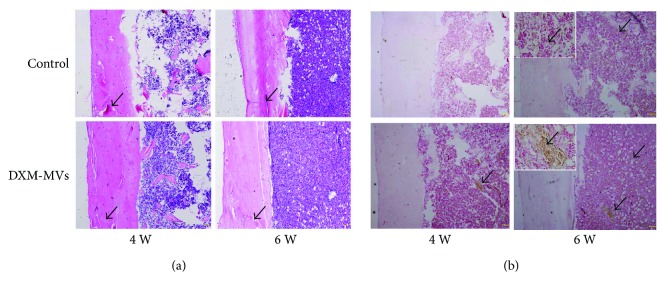
Histological analysis of the effects of DXM-MVs on bone healing at 4 and 6 weeks postsurgery. (a) Hematoxylin and eosin (H&E) staining of callus sections demonstrated accelerated bone regeneration in DXM-MV group. Arrows indicate the cortical gaps (scale bar, 200 *μ*m). (b) Alkaline phosphatase (ALP) was highly expressed at the defect sites of DXM-MV group. The insets are the images with larger magnification of the selected areas. Scale bar, 20 *μ*m. Arrows indicate the ALP-positive areas.

At the end of 4 or 6 weeks, histological examinations were performed to study the effect of DXM-MVs on bone regeneration. As shown in Figure 8(a), irregular fibrous callus could be found in the defect area in both groups on week 4 of postoperation, while the density and volume of these bone structures were lower in the control group. At 6 weeks after surgery, bone defect sites were completely filled with calluses in the DXM-MV group. The amount and area density of neo-formed bones were significantly increased when in comparison to the control group. Additionally, the ALP staining revealed significantly higher ALP activity in the DXM-MV group than that of the control group at the selected periods (Figure 9(b)). Thus, H&E and ALP data supported the in vivo findings.

## 4. Discussion

It is now well recognized that the biological functions of MSCs mostly rely on the activity of released MVs [29]. However, growing evidence suggest that the secretion and function of MVs are largely influenced by the surrounding environments of parent cells [13, 26, 30]. Numerous attempts have been performed to study the functional regulation of released MVs by varying the culture conditions. DXM is a synthetic form of glucocorticoid that has been found to cause osteoporosis or even pathological fracture under long-term administration [31]. Nevertheless, many studies have shown that DXM in vitro could enhance the osteogenic differentiation of MSCs accompanied by increased ALP activity and bone mineralization [18, 32]. In the present study, DXM was selected as a stimulus to be added into the MSC conditioned medium at varied dosages. The effect of DXM on the release and functions of MSC-MVs was studied based on physicochemical characteristics and their support of in vitro osteogenic differentiation and in vivo bone regeneration. We found that DXM had no evident impact on the release of MSC-MVs but played pivotal roles in regulating the biofunctions of MSC-MVs.

The MSCs under normal condition or DXM stimulation both produce MVs with the expected size range (100–400 nm) and with the typical spheroid-shaped morphology [33]. The concentration of MVs (normalized to the cell number) was almost at the same degree in the DXM-containing medium of stimulated MSCs to that of unstimulated cells. Additionally, the MVs collected from the different culturing conditions highly expressed the MSC-specific marker CD90, which confirmed the nature of these MVs. Altogether, the results of physicochemical studies revealed that the stimulation of DXM had no obvious effect on the release of MVs from MSCs.

Mediating the cell-to-cell communication has been regarded as one of the major function of MVs, which has attracted a great deal of interest in recent years [34, 35]. We examined whether MSC-MVs can interact with MC3T3 and how it is regulated by the DXM stimulation. Our results showed that the uptake of MVs by MC3T3 appeared to vary between DXM-MVs and n-MVs. The reason for this observation was not further investigated in this study, but, presumably, there might be a range of specific structural differences between the DXM-MVs and n-MVs, such as surface ligands or receptors, which may cause the differential interaction with the MC3T3. In addition, albeit speculative, the possibility could not be excluded that the carrying of the components such as proteins and miRNAs may differ among them. Further investigation is therefore necessary to address this difference.

Since the MVs isolated from DXM stimulated and unstimulated MSCs were shown to differentially interact with the MC3T3, we further examined whether the biofunctions of MVs were also different with respect to tailoring the subsequent cellular behaviors of MC3T3. We demonstrated that the DXM-MVs released from the DXM-stimulated MSCs largely increased MC3T3 proliferation and migration in a short culturing term as compared to the n-MV group, but dependent on the DXM dose. Moreover, the in vitro osteogenic differentiation experiments indicated that the calcium deposition and Runx2, ALP, and OPN were highly upregulated in MC3T3 treated with DXM-MVs (10^−7^ M). These findings seem to be contradictory to the report of Qin and the coworkers at a first view, who found that extracellular vesicles (EVs) derived from MSCs enhanced the osteogenic response of human osteoblasts [36]. However, the EVs are a family of vesicles such as apoptotic bodies, MVs, and exosomes which are different in definition and functions [36, 37]. Another potential reason for this observation could be attributed to the different recipient cells used.

MVs are submicron membrane fragments released from virtually all cell types and participated in regulating various functions of the target cells [38]. Notably, growing evidence demonstrates that environment stimuli largely affect the paracrine activity of parent cells and thus differ in regulating the protein production, gene expression, and behavior of recipient cells [12–14]. Herein, we verified that the MVs obtained from different stimuli were differentially interacted with MC3T3 and regulated the proliferation, migration, and osteogenic differentiation of MC3T3. Moreover, the biofunctions of MSC-MVs in regulating the cellular behaviors of recipient MC3T3 cells were largely dependent on the dose of the DXM. MVs extracted from DXM (10^−7^ M) stimulated MSCs' helped in maintaining MC3T3 functions and promoting the bone regeneration in vivo. It is well documented that DXM plays an important role in regulating the differentiation of MSCs into osteoblasts, which is largely dependent on the treatment dose and time [18, 39, 40]. Specifically, low DXM concentration enhances MSC commitment and facilitating differentiation [20, 21, 40] while high concentrations and long-term treatments suppress maturation and terminal osteoblast differentiation [22, 23]. Recently, Rimando and his coworkers found the dose and temporal regulation of DXM on MSC differentiation were correlated with the expression, degradation, and subcellular localization of glucocorticoid receptor (GR) in the MSCs, which is important in regulating gene transcription either through direct protein-protein interactions or facilitating the assembly of other regulatory proteins on the promoter regions of its target genes [40]. We assume here that the dose of DXM in the current study might also influence the genetic information (mRNA or microRNAs) of the target MSCs via GR receptors which act differently in regulating the cellular responses of MC3T3. Growing evidence suggests that MVs can act as a delivery system for the transfer of genetic information (mRNA and microRNAs) or can shuttle proteins to recipient cells [41]. Nevertheless, the potential functional RNAs or proteins delivered by the MVs in regulating the cellular behavior of MC3T3 have not elucidated in the current study.

## 5. Conclusion

DXM has no effect on the release of MVs from MSCs while influencing largely the functions of MSC-MVs. DXM-MVs enhance the osteogenic differentiation of MC3T3, accelerate expression of osteogenic genes and deposition of calcium phosphate, and promote bone formation in vivo. Overall, the current study represents a new strategy for the preparation of bioactive MVs for promoting bone regeneration.

## Figures and Tables

**Table 1:** Primers used for qRT-PCR.

	Forward primer	Reverse primer	Size
GAPDH	AAGGTCATCCCAGAGCTGAA	AGGAGACAACCTGGTCCTCA	196
Runx2	CCGCACGACAACCGCACCAT	CGCTCCGGCCCACAAATCTC	289
ALP	AACCCAGACACAAGCATTCC	GCCTTTGAGGTTTTTGGTCA	200
OPN	GACGATGATGATGACGATGG	CCTCAGTCCATAAGCCAAGC	195
